# SUR1-Associated Mechanisms Are Not Involved in Ischemic Optic Neuropathy 1 Day Post-Injury

**DOI:** 10.1371/journal.pone.0148855

**Published:** 2016-08-25

**Authors:** James D. Nicholson, Yan Guo, Steven L. Bernstein

**Affiliations:** 1 Department of Ophthalmology Visual Sciences, UMB School of Medicine, Baltimore, MD, United States of America; 2 Department of Anatomy and Neurobiology, UMB School of Medicine, Baltimore, MD, United States of America; Massachusetts General Hospital/Harvard Medical School, UNITED STATES

## Abstract

Ischemia-reperfusion injury after central nervous system (CNS) injury presents a major health care challenge with few promising treatments. Recently, it has become possible to reduce edema after CNS injury by antagonizing a sulfonylurea receptor 1 (SUR1) regulated ion channel expressed after injury. SUR1 upregulation after injury is a necessary precondition for the formation of this channel, and has been implicated in white matter injury after clinical spinal cord trauma. Glibenclamide, an SUR1 antagonist, appears to have neuroprotective effect against cerebral stroke in an open-label small clinical trial and great effectiveness in reducing damage after varied experimental CNS injury models. Despite its importance in CNS injuries, SUR1 upregulation appears to play no part in rodent anterior ischemic optic neuropathy (rAION) injury as tested by real-time PCR and immunohistochemical staining of rAION-injured rat optic nerve (ON). Furthermore, the SUR1 antagonist glibenclamide administered immediately after rAION injury provided no protection to proximal ON microvasculature 1 day post-injury but may reduce optic nerve head edema in a manner unrelated to ON SUR1 expression. Our results suggest that there may be fundamental differences between rAION optic nerve ischemia and other CNS white matter injuries where SUR1 appears to play a role.

## Introduction

Post-infarct CNS edema following sudden CNS ischemia-reperfusion injury has been shown to play an important role in increasing post-infarct damage, by increasing the damage penumbra after the initial insult [1–4]. Recently, SUR1 has been shown to associate with the transient receptor M4 channel (TRPM4) to form a non-specific monovalent cation channel sensitive to adenosine triphosphate (ATP) [5]. SUR1 is an ion channel regulatory protein that associates with TRPM4 and the inward rectifying potassium channel K_ir_6.x family to confer increased ATP sensitivity [5–8]. SUR1 expression is upregulated in injured tissue after focal ischemia-reperfusion injury. Continuous ion influx through this non-specific monovalent cation channel is postulated to cause cytotoxic edema in some cells and to result in progressive edema after injury. Inhibiting SUR1-mediated ion channel modulation with the small-molecule drug glibenclamide protects following CNS ischemia-reperfusion and traumatic brain function [2,3,8–10]. White matter edema following spinal cord trauma is also thought depend on SUR1-mediated ion channels [7]. Edema due to the SUR1-regulated TRPM4 cation channel is therefore believed to be a general feature of progressive edema-related CNS injury.

Nonarteritic anterior ischemic optic neuropathy (NAION) is an ischemic optic nerve injury. It is the most common clinical cause of sudden optic nerve (ON) related vision loss in individuals over the age of 50 [11]. Patients with NAION typically present with painless loss of vision associated with ON edema, a visibly prominent anterior ON swelling, ON ischemia and disruption of the normal nerve architecture. Later in the disease course, neuronal cell death, and permanent vision loss occur. Because the ON is a CNS white matter tract, we were interested in knowing if SUR1-related mechanisms were associated with the pathophysiology of this lesion.

Rodent anterior ischemic optic neuropathy (rAION) models were created to study the mechanisms of NAION damage and to evaluate potential NAION treatments [12–15]. These models express many of NAIONs stigmata, and have been shown to share many of the pathophysiological mechanisms associated with this disease, including capillary decompensation and progressive ON edema [16] leading to ON ischemia, inflammation and isolated retinal ganglion cell (RGC) loss [16,17]. Our prior studies showed that reducing ON edema and improving perfusion during the acute phase or rAION reduced long-term RGC death [16]. In the current study, we evaluated whether SUR1 upregulation is a driver of ON edema, and whether the SUR1 antagonist glibenclamide is effective in reducing the damage associated with sudden ON ischemia.

## Methods

### rAION induction and tissue collection

This study was carried out in strict accordance with the recommendations in the Guide for the Care and Use of Laboratory Animals of the National Institutes of Health. The protocol was approved by the Animal Care and Use Organization (ACUO) of the University of Maryland at Baltimore (#0908001). rAION induction was generated as previously reported [14,15]. Intravenous (i.v.) Rose Bengal (RB) (Sigma-Aldrich; 2.5 mg/kg 0.20 μm sterile filtered in saline) was administered intravenously. The intraocular portion of the ON was visualized using a contact lens. RB was activated by intraocular optic nerve illumination via 532nm wavelength laser light (OccuLight GL medical laser system, Iris Medical) by illuminating the optic disc with a 500 μm spot size/50mW intensity (rat). This treatment results in capillary decompensation at the ON head, and progressive ON edema that peaks 1–2d post-induction. This injury results in a ~55% loss of retinal ganglion cells by 30 days post-induction [14,15,17]. Animals were examined after receiving i.p. ketamine/xylazine (80 mg/kg / 40 mg/kg) anesthesia and euthanized by i.p. pentabarbitol overdose. ONs were removed from 3 animals at 1 day post-rAION, fixed in 4°C 4% paraformaldehyde overnight, placed in 70% ethanol and paraffin embedded. Nerves were sectioned longitudinally at 7 μm thickness.

### In vivo imaging

ONH edema was evaluated using a thin plano-concave contact lens that enables visualization of the rodent retina and optic nerve head. The retinal cell layers and ONH diameter were imaged via spectral-domain optical coherence tomography (Spectralis®, Heidelberg Instruments) using the contact lens.

### Ribonucleic acid (RNA) isolation and quantitative real-time polymerase chain reaction (qRT-PCR)

The proximal 3 mm of the ON was surgically isolated after removing the eye and stored at -80°C. Nerves were crushed over dry ice, homogenized in lysis buffer, and total RNA was isolated using the RNeasy micro kit (#74004, Qiagen Inc.) using linear acrylamide (Ambion Inc.) as a carrier and DNAse-1 treated to eliminate genomic DNA contamination., followed by proteinase K digestion and chloroform extraction. RNA was analyzed for purity and quality using an Agilent Bio-analyzer. Because of the low yield for individual ON samples, the single chimeric primer amplification method (SPIA-Ovation pico system, Nugen Corp.) was used to provide non-biased linear amplification of small amounts of mRNA. This reduced animal use, enabling comparison of gene response in individual rats rather than using pooled mRNA. RNA was converted to first strand random primed cDNA via RETROscript 1710 kit (Ambion Inc.) and used for qRT-PCR with gene-specific primers, using Syber green dye (Bio-Rad Laboratories) for detection, with cyclophilin B used as a reference gene. Specific mRNA levels were evaluated using the ΔΔCT method of difference subtraction from control gene expression levels. The following primers were used: TNFα (f) actcccagaaaagcaagcaa, (r) cgagcaggaatgagaagagg; IL-1β (f) gctagggagcccccttgtcg, (r) gctctgagagacctgacttggca; cyclophilin B (f) tgacggtcaggtcatcactatc, (r) ggcatagaggtctttacggatg.

### ON vascular filling and quantification

Rodent ON vascular filling was performed following Nicholson *et al* (2012) [16]. Quantitative ON vascular analysis was performed using tissue from terminally anesthetized rats. After euthanasia, animals were placed on a warming pad (~38°C) and transcardially perfused sequentially with the following heated (~38°C) solutions: 120 ml heparinized saline (50 units/ml) with 2 μg/ml atropine sulfate (Sigma Chemicals) and 100 μM adenosine (HAAS solution), 50 ml fluorescein-conjugated bovine serum albumen (FITC-BSA) with 2% dissolved gelatin (300 bloom, Sigma Chemicals) in HAAS solution, 20 ml FITC-BSA with 4% dissolved gelatin in HAAS solution. Tissues were fixed in 4% neutral-buffered paraformaldehyde (PFA), washed in phosphate buffered saline (PBS), embedded in 10% gelatin (#G1890, Sigma), and re-fixed for an additional 24 h in PFA. Tissues were cryosectioned to 40 μm and imaged by confocal microscopy using tiled z-stacks imaged on a Zeiss LSM510 Duo (Carl Zeiss Microscopy, Gottingen) fitted with a 40x 1.4 NA oil objective. Vascular data was quantified using a filament model constructed using Imaris software (Bitplane Software) (See Nicholson *et al*. (2012) for method details [16]).

### Glibenclamide delivery via Alzet Pump Implantation

Rats were anesthetized with ketamine and xylazine as described in the rAION method and remained under anesthesia with the addition of subcutaneous 0.25 mL of 0.12% bupivacaine local anesthetic during pump implantation. Body temperature was maintained using a thermostatically controlled Gaymar heating pad (38°C). A subcutaneous osmotic pump (Alzet Cat #2001, Durect Corp.) containing glibenclamide or vehicle was inserted through a ~1 cm incision in the nape of the neck. The wound was closed with surgical staples. An intraperitoneal (i.p.) loading dose of 10 μg/kg glibenclamide in DMSO or DMSO vehicle (1 mL/kg) was then administered. The rats recovered in a clean cage under thermal control of the Gaymar heating pad (Gaymar Industries Inc., Orchard Park, NY). Osmotic pumps and loading dose syringes were prepared and randomly coded according to a recent protocol [9]. The osmotic pumps were primed overnight at 37°C as per the manufacturer’s instructions in order to deliver a constant dose once implanted.

### Preparation of Brain Tissue

Mouse brains from SUR1(-/-) mice on a C57BL/6 were obtained from a colony at the University of Maryland Baltimore. Mouse brain tissue from WT C57BL/6 mice was used as wild-type tissue. In both cases, mice were euthanized with CO_2_ and perfused with 4% PFA. Whole brains were extracted and fixed for 2 days in 4% PFA, then sunk in 30% sucrose (Ultrapure, Sigma) in PBS for 2 days. The forebrain was manually sectioned with a razor blade to expose a coronal section that included both the hippocampus and choroidal tissue. The brain was then placed in 70% ethanol solution and paraffin embedded. Brains were sectioned via microtome to 7 μm thickness and annealed onto Superfrost slides (Fisher Scientific) overnight at 50°C.

### SUR1 Immunohistochemistry

Sections were deparaffinized by immersing in xylene (Sigma Cat #247642) for 2 minutes followed by an additional 10 minutes immersion into fresh xylene. The use of a long second immersion in clean xylene is essential for good staining. The sections were rehydrated in graded ethanol solutions (100%, 95%, 80%, 50%) for 2 minutes in each solution, then transferred to PBS. In the following steps, it is essential that no detergent be used. The addition of Triton X-100 caused irregular as well as diffuse background staining and resulted in false positive staining. No antigen retrieval was used. For rodent tissue with Ni-DAB visualization, endogenous peroxidase was inactivated by treating with 1% H_2_O_2_ + 10% MeOH in PBS for 15 minutes then washed in PBS changed 6x over 30 minutes. Endogenous biotin was blocked by incubating the slides in 1:1000 streptavidin-Cy3 (Jackson Immunoresearch Labs) for 1 hour, washing 4x over 1 hour in PBS, blocking in 2% normal donkey serum made in a saturated solution of biotin in PBS (Cat# B4501, Sigma) for 1 hour, then washing 6x in PBS over 1 hour. SUR1 was detected by incubating slides in the following solutions: 1:2000 rabbit anti-SUR1 antibody (SC-25683, Santa Cruz Biotechnology) in PBS overnight at room temperature, 6x wash in PBS over 1 hour, 1:2000 biotinylated donkey anti-rabbit secondary antibody (Jackson Immunoresearch) in PBS for 35 minutes, wash 6x in PBS over 1 hour, streptavidin-peroxidase (Vectastain Elite ABC Kit, Vector Labs) for 1 hour, wash 6x over 1 hour in PBS, equilibrate in 0.175 M sodium acetate. The slides were stained with nickel-DAB precipitate by incubating in the following solution: 50 ml 0.175 M sodium acetate, 1.25 g nickel sulfate hexahydrate, 1 DAB tablet (Cat #D5905, Sigma Chemical Co.) dissolved in 0.175 M sodium acetate using sonication. The developing reaction was stopped by rinsing 3x in 0.175 M sodium acetate. The rinse solutions and all reagents containing DAB were collected and disposed of safely. The slides were then equilibrated in PBS, dehydrated through an ethanol gradient into xylene, and mounted with a coverslip using DPX mounting media (Sigma). Nuclear counterstaining was done using 15 minute exposure to 0.1 μM 4',6-diamidino-2-phenylindole (DAPI) in PBS before mounting in an anti-fade mounting media.

### Real-time PCR

ON mRNA was prepared as above. A SUR1 primer set was selected from a previously published paper; (f) tgaagcaactgcctccatc, (r) gaagcttttccggcttgtc [18]. The primer set was optimized for temperature by performing PCR reactions on rat brain cDNA across a temperature gradient 57–62°C and examining the reaction products on a DNA gel for the presence of a single product band. The optimum temperature for a single band was 59°C. cDNA was prepared from mRNA (described previously) extracted from the first 3 mm of individual rat ON at 1 day post-rAION with the OD subject to rAION and the OS not subject to rAION. mRNA samples were not pooled. Two treatment conditions were examined: 100 μg/kg 15d-PGJ_2_ *vs*. vehicle. The mRNA from individual rats (n = 4) was reverse transcribed to cDNA and subjected to linear amplification using the SPIA kit (SPIA-Ovation pico system, Nugen Corp.). All cDNA was diluted 1:10 in water before qRT-PCR testing. We used cyclophilin B as our housekeeping control gene. Primers were: (f) tgacggtcaggtcatcactatc, (r) ggcatagaggtctttacggatg.

## Results

### Continuous administration of glibenclamide via osmotic pump did not improve vascular perfusion 1 day after rAION

We tested SUR1 involvement in the acute ischemic phase of rAION injury progression by inducing a rAION lesion in the right eye of 19 male Sprague-Dawley rats. The uninjured left eye of each animal served as an internal control. rAION-injured rats received either glibenclamide (n = 10) or vehicle (n = 9) via a combination of intraperitoneal (i.p.) injection and subcutaneously-implanted osmotic pump administered immediately post-injury. Glibenclamide administered in osmotic pumps enabled maintenance of a constant plasma concentration over a 1 day period. The glibenclamide serum concentration produced by this protocol was previously determined to be ~5 nM with a maximal concentration not exceeding 10 nM [9].

**Fig 1:**
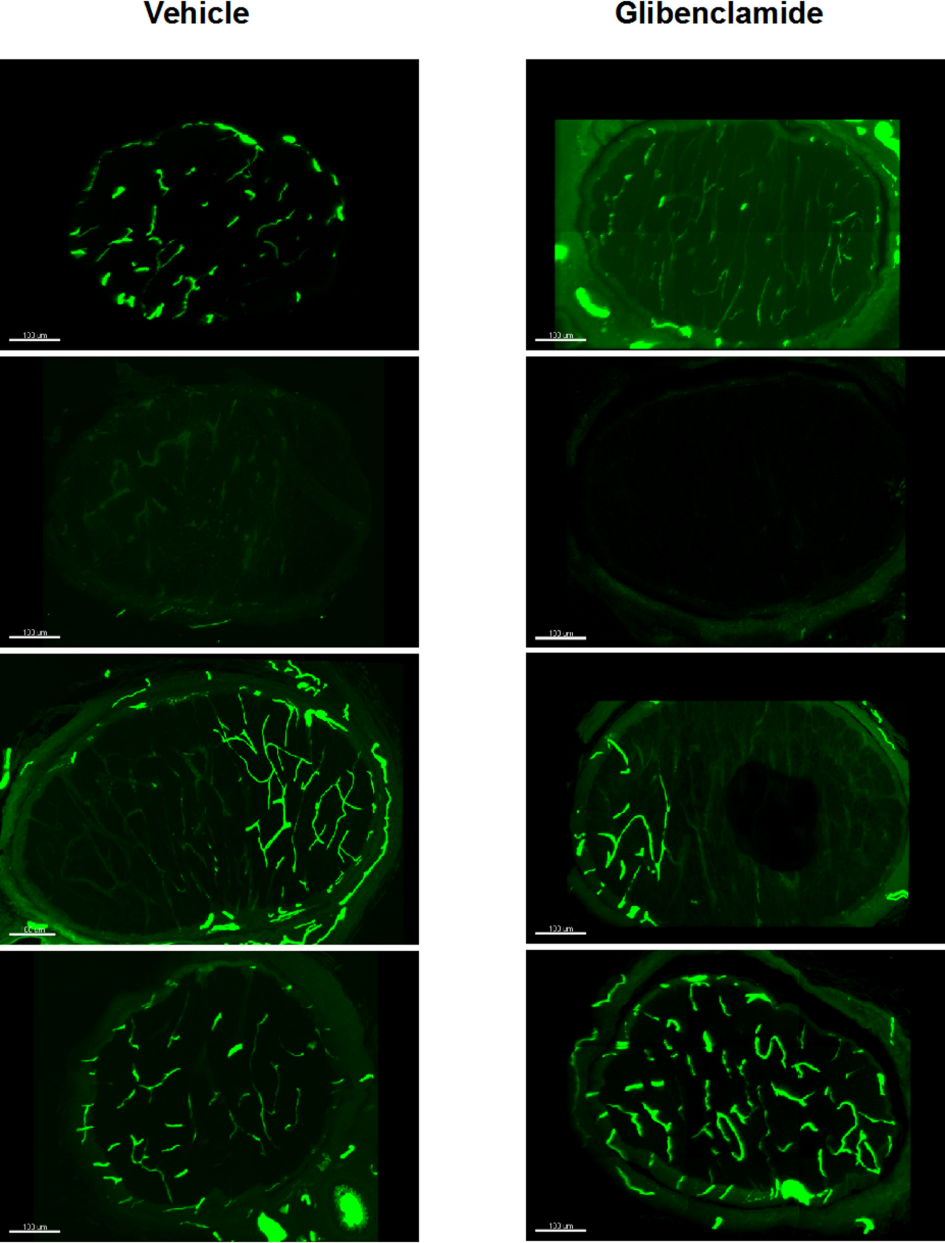
Fluorescent capillary filling of ischemic ON at 1 day post-rAION after glibenclamide or vehicle treatment. Rats were treated via implanted osmotic pump and i.p. loading dose of either vehicle or glibenclamide. Images show injured ON 1 day post-rAION in maximum intensity projection of confocal microscope z-stacks where vessels were perfused with fluorescent gelatin [16]. Injury severity ranged from complete loss of perfusion to limited sectorial dropout of perfusion. Previously, we showed a marked improvement in ON perfusion when rAION was treated with a single injection of prostaglandin J_2_ [16]. While we cannot conclude glibenclamide has no effect, this drug does not appear to provide reliable protection against early loss of microvascular perfusion in rAION. For both treatment groups, n = 4. Scale bar = 100 μm.

We quantified the proximal ON microvascular perfusion of glibenclamide-treated rats compared to vehicle-treated rats at 24 hours post-rAION using fluorescent gelatin perfusion of ON microvasculature (previously described in Nicholson et al (2012) [16]). Glibenclamide treatment did not improve proximal ON perfusion compared to vehicle (21±18% Glibenclamide, 37±26% Vehicle, p>.05, Student’s t test, Prism, Graphpad Inc.) seen by quantifying fluorescent gelatin perfused ON at 1 day post-rAION with vehicle or glibenclamide treatment (see Fig 1 for perfusion of rAION ONs). Due to a high rate of failure for the capillary filling procedure, the ON yield allowed only n = 4 for each group. The failure of procedure rate was higher with pump-implanted animals (58% or 11/19) than for the immediately preceding 20 animals used in our prior study (30% or 6/20) [16].

Given the high failure rate of vascular filling, we performed a post-hoc power analysis that revealed a group size of 13 would have been required to have a 95% probability of achieving a significant result, given the 0.71 effect size of the observed glibenclamide *vs*. vehicle groups (using Cohen's d statistic with α = 0.05 in G*Power 3 Software [19]). By comparison, the effect size of perfusion volume from a previous study comparing rAION injury in vehicle-treated to 15d-PGJ_2_-treated animals had a much larger Cohen’s d of 2.85 and showed protection by 15d-PGJ_2_ [16]. Given the 58% average failure of procedure rate found with the osmotic pump-treated animals, an experimental group size of approximately n = 49 would have been required to have a 95% probability of achieving a significant result, which is an excessive number of animals given the modest effects found. Additional animals were not added to these groups because other observations showed that SUR1 is not upregulated 1 day post-rAION.

### Glibenclamide treatment did not reduce ONH edema relative to vehicle treatment as determined by spectral-domain optical coherence tomography (SD-OCT)

**Fig 2:**
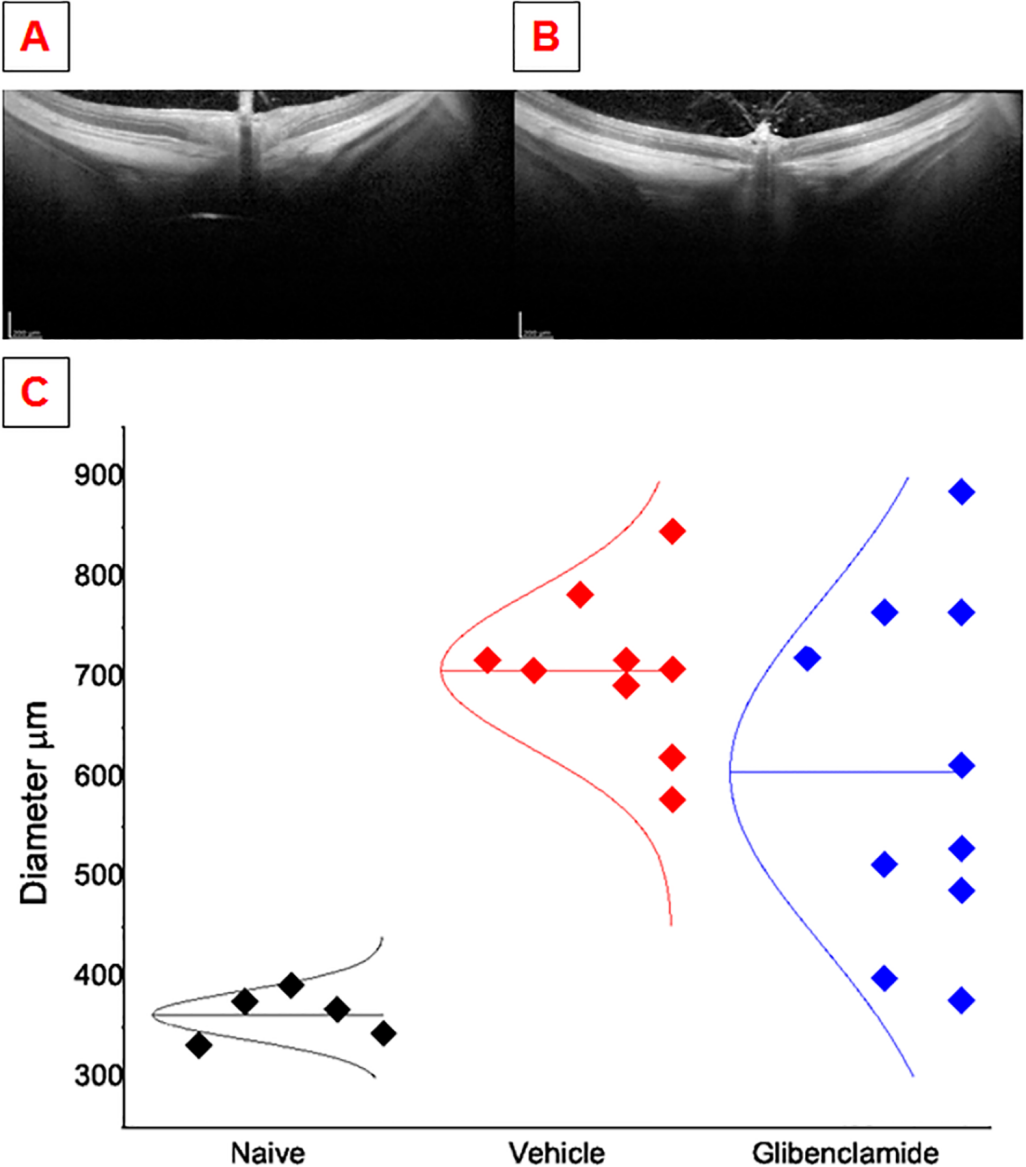
OCT comparison of rAION injured ONH treated with glibenclamide vs. vehicle. Panels A and B are typical images of rAION at 1 day post-injury treated with vehicle and glibenclamide, respectively. Panel C shows ONH edema measurements across rat ONH in naive rats compared with the edematous diameter with either glibenclamide or vehicle treatment. A normal curve is plotted over each category of data to indicate the mean and variance of the data.

To assess protection that may occur through reduction of ONH edema independent of ON microvascular protection, we examined the extent of ONH edema using SD-OCT imaging, testing for glibenclamide-associated edema reduction in the same groups used for the microvascular experiments, with an additional 2 animals that were not perfused for vascular quantification. Typical SD-OCT images from a 1 day rAION-injured ON are shown in Fig 2A and 2B (vehicle-treated and glibenclamide-treated, respectively). The edematous area of the vehicle-treated rAION-induced ONH (Fig 2A) can be seen to obscure the laminar structure of the retina. We quantified the extent of the injury by measuring the diameter of the edematous area across the ONH (from inner plexiform layer to inner plexiform layer) at the level of the hyaloid artery (frequently visible in OCT images as a protrusion from the ON) in glibenclamide-treated (n = 10) and vehicle-treated groups (n = 9). The mean edematous diameter of the glibenclamide-treated animals was smaller than the group mean diameter of vehicle-treated. The effect of ONH edema reduction by glibenclamide is modest. This is supported by the large standard deviation and modest reduction in edema (605+/- 54 μm for glibenclamide vs 708 +/- 26 μm for vehicle) that glibenclamide provides (p = 0.06, single tailed Student's t test, Prism, Graphpad Inc.). A single-tailed t test is appropriate due to the hypothesis proposing only to test for reduced edema and because the entire ONH is typically edematous 1 day after rAION, making it very unlikely that ONH edema can be increased without affecting retinal circulation. The ONH diameter of uninjured eyes (362±24 μm) is shown for comparison.

We also examined OCT data from the same groups of animals using a subjective examination for improvement of ONH edema. Three researchers blinded to the treatment condition were asked to rank order the severity of the edema based upon a mutually agreed upon scale of edema severity which included such features as the appearance of ON edema, expansion of the ONH into the globe and presence of subretinal fluid (fluid trapped between the outermost layer of the retina and the choriocapillaris). All three researchers found no gross difference between the two treatment groups (p>0.05, Mann-Whitney U test, Prism, Graphpad Inc.). Thus, there is no evidence that reduced rAION-induced ONH edema using either a subjective or quantitative test.

### ON SUR1 is not upregulated 1 day after rAION

ON mRNA was isolated from the initial 3 mm of ON and subjected to linear amplification. Injured OD (right) ONs were compared to uninjured OS (left) ONs (n = 4). ON SUR1 mRNA was not significantly upregulated in response to 1 day rAION by qRT-PCR using the ΔΔC_t_ method (ΔC_t_ vs. CycB, injured 9.08±1.16 vs. uninjured 9.16±0.86, p > 0.05 Student’s t-test).

**Fig 3:**
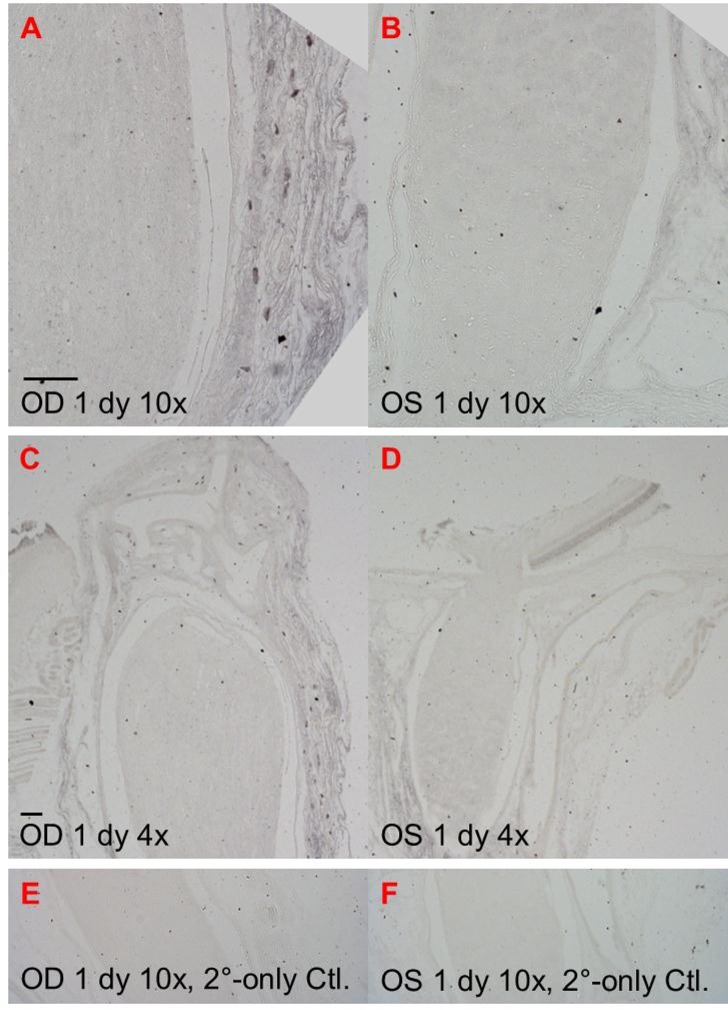
SUR1 staining in rat ON after rAION. Panels A & C show 1 day post-rAION (OD) longitudinally sectioned ON at different magnifications. Panels B & D show different magnification of the uninfarcted ON (OS) from the companion eye to the injured ON. Panels E & F show secondary-only controls from the same animals demonstrating the lack of non-specific background staining for both injured and non-injured ON. Scale bars are 100 μm. OS & OD are standard notation for left eye and right eye, respectively.

To be certain that SUR1 upregulation was not isolated to a small number of ON cells that might not be detectable via RT-PCR, we immunostained three sets of rat ONs (uninjured and 1 day post-rAION) for SUR1, where the animals received the i.v. vehicle treatment previously used to test 15d-PGJ_2_ [16]). Rats were euthanized 1 day post-rAION. All nerves were sectioned longitudinally to show both the proximal ON and ONH. SUR1 immunohistochemical staining (imaged using Ni-DAB chromagen) showed no increased signal in the substance of the rAION-injured ON *vs*. the uninjured ON, suggesting that SUR1 upregulation does not occur in the rAION-injured ON (Fig 3 panels A *vs*. B (medium magnification) and C *vs*. D (low magnification)), although some large SUR1-positive cells localized to the injured ON sheath which are possibly marginating neutrophils or SUR1-expressing endothelial cells (Fig 3 panels A & C). A positive control staining (brain choroid plexus from C57Bl/6 mouse) and negative control (brain choroid plexus from SUR1-/- mouse on C57Bl/6 background) were performed (data not shown) confirmed the specificity and appropriate concentration of the Santa Cruz rabbit anti-SUR1 antibody used for immunohistochemistry.

Thus, while SUR1 may be present in rat ON, the methods we used to observe SUR1 expressions after rAION did not show the strong SUR1 upregulation that was seen in other types of CNS ischemia. This lack of ON SUR1 upregulation is consistent with the lack of effectiveness of the SUR1 antagonist glibenclamide against rAION-induced ON edema demonstrated above.

## Discussion

We examined the role of SUR1 in exacerbating the ischemic phase of rAION injury. Our recent studies have shown that following rAION, the anti-inflammatory compound 15d-PGJ_2_ given i.v. or intravitreally within 5 hours post-injury can reduce early ON edema, providing microvascular protection at 1 day [16,20]. This protection is also seen in the primate model of NAION [21]. In contrast, the SUR1 antagonist glibenclamide failed to provide early microvascular protection. Furthermore, glibenclamide’s molecular target SUR1 was not upregulated after rAION by 1 day. Since SUR1 upregulation after traumatic injury and major ischemic events has been shown to be an important factor in producing CNS edema and damage [2,6,8,9,22], our findings support the hypothesis that upregulation of SUR1 expression is not involved in post-rAION ON edema formation. Further research may be warranted to study SUR1 upregulation up to 7 days post- rAION to assess its role in delayed edema.

Since glibenclamide can reduce circulating blood glucose levels, we were careful to maintain blood glibenclamide below 10nM [22]. This low level of glibenclamide administration prevents pathologic reduction in blood glucose levels. Also, Glibencamide’s EC_50_ for PPARγ activation is three orders of magnitude higher than the dose received by our rAION animals.[23] Thus, neither glibenclamide-induced hypoglycemia nor PPARγ activation were considered to be factors in interpreting our results.

Interestingly, glibenclamide appeared to provide some degree of reduction of ONH edema post-rAION. This result approached, but did not attain statistical significance (p = 0.06). Reduction of ONH edema is widely suspected to benefit ON ischemia due to reduction of a proposed compartment syndrome in the ON [11,24]. Reduction of ONH edema without a concomitant reduction in ON ischemia or neuroprotection suggests that there may be additional factors in development of NAION pathology beyond the simple mechanism of vascular compartment syndrome that has been proposed for the clinical disease. Other possibilities for reduction of ON edema without reduction in ultimate outcome may include blockade of vascular relaxation by an adenosine-associated mechanisms. Glibenclamide has been shown to inhibit adenosine-mediated retinal vasodilation with doses as low as 5 nM [25–28]. This occurs by interacting with the ATP-sensitive potassium (K_ATP_) channel localized in retinal vessel pericytes [29]. Since our animals received an <10 nM continuous glibenclamide dose, this may be sufficient to block adenosine-mediated retinal vasodilation, and potentially reduce ONH edema. Such a mechanism would be consistent with our current observation that the SUR1-glibenclamide target was not strongly expressed in rodent optic nerve under resting conditions or after rAION. Thus, it may be worthwhile to re-examine a potential role for glibenclamide for treatment of ophthalmic conditions where vascular dilation-associated ONH edema itself is pathological.

Another explanation for the lack of SUR1 involvement in rAION *vs*. other ischemic injury models may be that rAION injury results in a much slower change in intracellular ATP concentration, relative to the rapid drop in intracellular ATP concentration seen after MCAO or TBI [30]. Our previous study demonstrated that endothelial cell swelling and reduction of microvascular perfusion were important characteristics of early rAION [16]. We speculate that endothelial cells undergoing a slow change in extracellular environment in our model injury may not experience significant SUR1 upregulation as is found in sudden ischemia models where glibenclamide has shown great effect.
